# Commentary: Plasma thrombin-antithrombin complex as a candidate biomarker for coronary slow flow

**DOI:** 10.3389/fcvm.2025.1682454

**Published:** 2025-10-16

**Authors:** Ramazan Astan

**Affiliations:** Department of Cardiology, Batman Training and Research Hospital, Batman, Türkiye

**Keywords:** coronary slow flow, TIMI frame count, coronary microvascular dysfunction, coronary angiography, biomarkers

A Commentary on Plasma thrombin-antithrombin complex as a candidate biomarker for coronary slow flow By Mo J-h, Liang B, Cen J-t, Li W-y, Mo Y-a, Tang M-c, Ye D, Long Q-x, Hu X and Zhai Y-s (2025). Front. Cardiovasc. Med. 12:1621655. doi: 10.3389/fcvm.2025.1621655

## 1 Introduction

I read with great interest the article by Mo JH et al. titled “Plasma thrombin-antithrombin complex as a candidate biomarker for coronary slow flow” recently published in Frontiers in Cardiovascular Medicine (1). The authors present compelling evidence that plasma thrombin-antithrombin (TAT) complex levels are significantly elevated in patients with coronary slow flow (CSF), proposing it as a potential non-invasive biomarker. While the findings are noteworthy, we would like to raise several methodological concerns that we believe warrant further clarification.

## 2 Inconsistency and ambiguity in CSF definition and grouping

A central concern lies in the apparent inconsistency and ambiguity surrounding the definition and operationalization of CSF within the study. In the methodology section, the authors describe calculating an average TIMI Frame Count (TFC) by summing the corrected TFCs (CTFC) of the LAD, LCX, and RCA and dividing by three a method aligned with literature characterizing CSF as a diffuse microvascular disorder. However, in the same section, the authors also define CSF as “TFC >27 in at least one coronary artery,” which reflects a segmental approach.

Further compounding this issue, Table 1 presents both average TFC values and artery-specific TFCs (LAD, LCX, RCA). However, it remains unclear whether patients were classified based on average TFC, single-vessel TFC, or a combination of both. For example, a patient with an average TFC below the pathological threshold but a single artery (e.g., RCA) slightly above 27 may have been included in the CSF group. The concurrent mention and presentation of both methods without a clear statement of which criterion was applied creates confusion, introduces potential heterogeneity in patient classification, and limits reproducibility.

## 3 Fixed threshold of TFC >27 for all arteries

The application of a uniform threshold of TFC >27 to define CSF across all three major coronary arteries may not be appropriate given their differing anatomic lengths and flow characteristics. According to Gibson et al. (2), the normal unadjusted TFC for LAD is approximately 36, and to account for its longer length, it is conventionally divided by 1.7, resulting in a corrected TFC (CTFC) of ∼21.2. Some studies propose a pathological cut-off of >27.6 for LAD CTFC, corresponding to approximately two standard deviations above the mean.

This distinction is critical because the same degree of statistical adjustment is not typically applied to the LCX or RCA. These arteries have shorter and more consistent lengths, with average unadjusted TFC values of approximately 22 and 20, respectively. Therefore, no correction factor like the LAD's 1.7 is necessary. Instead, abnormal thresholds for LCX and RCA (e.g., >22.6 and >20.4, respectively) are derived from either upper limit statistics (mean + 2SD) or receiver operating characteristic (ROC) curve analysis. Applying a single uniform cut-off value (i.e., >27) across all vessels disregards these anatomical and statistical nuances and risks misclassification, particularly by overestimating CSF prevalence in LAD or underestimating it in RCA.

## 4 Lack of information on contrast injection parameters

TFC measurements are known to be sensitive to technical factors such as contrast injection speed, volume, and method. The study does not specify whether contrast was administered manually or via power injector, nor does it provide details regarding injection rate or total volume. These factors can directly affect the number of frames required for contrast to reach distal landmarks, influencing TFC results and the diagnosis of CSF. Future studies should control for or at least report these parameters to enhance methodological transparency.

In conclusion, while the association between elevated TAT complex levels and CSF is promising, the aforementioned ambiguities in diagnostic criteria and methodology warrant clarification. Clear and consistent definitions are essential to ensure reproducibility, accurate classification, and the clinical applicability of novel biomarkers. We commend the authors for their valuable contribution and hope that these considerations will further strengthen research in this area.
